# HLA DRB1*03 as a possible common etiology of schizophrenia, Graves’ disease, and type 2 diabetes

**DOI:** 10.1186/s12991-017-0128-4

**Published:** 2017-02-02

**Authors:** Aicha Sayeh, Cheker Ben Cheikh, Ali Mardessi, Meriem Mrad, Brahim Nsiri, Abdelaziz Oumaya, Najiba Fekih-Mrissa

**Affiliations:** 1 Laboratoire de Biologie Moléculaire, Service d’Hématologie., Hôpital Militaire de Tunis, Montfleury, 1008 Tunis, Tunisia; 2 Faculté des Sciences de Tunis, Université Tunis el Manar, 2092 Tunis, Tunisia; 3 Service de Psychiatrie, Hôpital Militaire de Tunis, Montfleury, 1008 Tunis, Tunisia; 4 Faculté de Médecine de Tunis, Université de Tunis El Manar, 1007 Tunis, Tunisia; 5 Service Oto-rhino-laryngologie, Hôpital Militaire de Tunis, Montfleury, 1008 Tunis, Tunisia; 6 Académie Militaire Fondouk Jédid, 8012 Nabeul, Tunisia

**Keywords:** Graves’ disease, Schizophrenia, Type 2 diabetes, HLA, Autoimmunity

## Abstract

**Background:**

Autoimmune diseases and schizophrenia share many common features. Association studies confirm a shared genetic association in the human leukocyte antigen (HLA) region between schizophrenia and most autoimmune diseases. To our knowledge, the simultaneous syndromes of Graves’ disease (GD) and type 2 diabetes (T2D) in schizophrenia are rare in Tunisia.

**Case presentation:**

We report a case of a 42-year-old woman admitted to the department of psychiatry for an acute relapse of chronic schizophrenia. Her medical history revealed that she was followed for Graves’ disease and for a type 2 diabetes mellitus. A low-resolution HLA typing was performed by polymerase chain reaction sequence-specific primer (PCR-SSP) techniques according to determine the patient’s haplotype.

**Conclusions:**

Our study suggests that the HLA DRB1*03 allele may explain a common etiology underlying the co-morbidity of Graves’ disease, type 2 diabetes, and schizophrenia in our patient.

## Background

People with schizophrenia often develop somatic symptoms, particularly autoimmune disorders [1]. Previous studies have reported that, compared with the general population, individuals with schizophrenia have a different prevalence of some autoimmune diseases and experience different immunological responses [1, 2]. Autoimmune involvement may have potential influence on central nervous system (CNS) pathologies which generates antibodies against the brain tissue in those with schizophrenia [3, 4]. A pathogenic theory of schizophrenia posits its proximate cause to be an infection during early development whereby antigens, similar to that found in CNS tissue, give rise to auto-antibodies that act against the brain.

Dopamine, which is inhibitory to TSH secretion, was found to be increased in schizophrenic patients. Subsequent clinical evidence has also shown an excess of thyroid hormone dysfunction in schizophrenia that can result in cognitive function impairment with emotional and behavioral disturbances. Furthermore, other studies have revealed an association between Graves’ disease and schizophrenia. Some have attributed these manifold observations to dysfunction of the hypothalamus-pituitary-thyroid axis [5–7].

### Case presentation

A 42-year-old woman was admitted to the Department of Psychiatry of the Military Hospital of Tunis for an acute relapse of chronic schizophrenia. Her medical history revealed that the patient was followed for seven years for Graves’ disease treated by synthetic anti-thyroids and for a type 2 diabetes mellitus treated by metformin. The patient received antipsychotic therapy for three weeks during recovery in the Department of Psychiatry and is now followed regularly for her associated diseases. The co-existence of those diseases has motivated a genetic analysis to seek a common syndromic pathology. The patient gave informed consent to giving a blood sample for genetic analysis. The study protocol was approved by the Ethics Committee of the Military Hospital of Tunisia and has therefore been performed in accordance with the ethical standards laid down in the 1964 Declaration of Helsinki and its later amendments.

### HLA typing by DNA amplification

Genomic DNA was extracted from peripheral blood samples of patients and healthy individuals using the QIAamp DNA Blood Mini Kit (QIAGEN GmbH, Hilden, Germany). Low-resolution HLA typing was performed by polymerase chain reaction sequence-specific primer (PCR-SSP) techniques according to Micro SSP DNA Typing Trays DRB/DQB (One Lambda Inc. Canoga Park, California, USA). Amplified DNA fragments were detected by agarose gel electrophoresis (2.5% agarose gel), stained with ethidium bromide, and UV transillumination. One Lambda DNA/LMT software version 3.98 was used to detect specific DRB1 and DQB1 alleles.

## Results

The genetic investigation revealed that the patient carried the HLA DRB1*03-HLA DQB1*02 haplotype. We suppose that HLA DRB1*03 may be the common cause of these three simultaneous diseases.

## Discussion

We report a case of a patient presenting co-occurrences of Graves’ disease, type 2 diabetes, and schizophrenia. The genetic investigation revealed that the patient carried the DRB1*03-DQB1*02 haplotype. Many subsequent studies have noted similarities between schizophrenia and other autoimmune diseases [1]. Firstly, the diagnostic procedures are largely dependent on clinical criteria such as the lack of a specific etiological diagnosis. Secondly, the treatments are usually palliative, not curative. Thirdly, both are complex disorders caused by multiple genetic, environmental, and lifestyle factors, and the interaction between these factors do not follow a clear Mendelian pattern of inheritance. Furthermore, due to limited knowledge of pathophysiology, individuals with schizophrenia and/or certain autoimmune diseases may be similar in clinical presentation, but their underlying etiologies are very heterogeneous.

Recent genome-wide association studies have identified a growing number of disease-associated loci and have provided enormous insight into the etiology of complex genetic disorders, including schizophrenia and autoimmune diseases [8, 9].

Strikingly, consistent with the results from previous linkage and case–control association studies, genome-wide association study results confirmed a shared genetic association in the human leukocyte antigen (HLA) region between schizophrenia and most autoimmune diseases. Caucasian patients with GD usually present the HLA-DRB1*03 allele in strong linkage disequilibrium with the HLA-DQB1*0201 [10, 11]. Also, in native South Africans, HLA-DR1 has been associated with GD [12]. Moreover, very few studies examined a possible role for HLA class II in type 2 diabetes in relation to autoimmune markers and latent autoimmune diabetes in adults and association with complications of diabetes. This was exemplified by the findings that DQB1*0201 and DQB1*0302 [13], DRB1*03/04-DQB1*0302 [14, 15], and DRB1*03-DQA1*0502-DQB1*0201 (DR3-DQ2) were positively associated with type 2 diabetes [16]. Furthermore, the HLA-DRB1 gene has been a focus in investigating the HLA association with schizophrenia [17]. A strong positive association between HLA-DRB1*03 and HLA DQB1*02 alleles and schizophrenia has widely been reported in the Saudi and Tunisian populations [18, 19].

Although associations between the HLA loci and GD, schizophrenia, and T2D are well established, the underlying mechanisms through which HLA molecules confer susceptibility to those diseases simultaneously are still unclear. It is known that the major function of HLA class II molecules (DR, DQ, and DP), in the normal cellular and humoral immune response, is to bind peptide antigens and present them to T cell receptors [20]. There is persuasive evidence that these diseases are associated with specific amino acid sequences of the DRB1 and DQ genes. It is thought that different HLA alleles have different affinities for peptides from auto-antigens such as the TSH receptor, dopamine receptors, and GAD antibodies. Knight postulates that the acute positive symptoms of schizophrenia are caused by auto-antibodies which interact with and stimulate dopamine receptors in certain neural pathways of the brain (dopamine-receptor-stimulating auto-antibodies) [21]. Despite the failure of some studies to detect dopamine receptor auto-antibodies in schizophrenia patients [22, 23], the hypothesis of a major role of dopamine receptor serum antibodies is further supported by recent findings of an increased production of pro-inflammatory cytokines and auto-antibodies in acutely ill schizophrenia patients [24, 25]. These antibodies would be analogous to the thyroid-stimulating auto-antibodies which cause the hyperthyroidism of Graves’ disease [21]. In the same way, GAD antibodies would also be analogous to both previously discussed antibodies. Thus, only specific HLA-DR alleles with the appropriate amino acids in its peptide-binding pocket would allow an auto-antigenic peptide to bind and to be recognized by the T-cell receptor [26]. This will eventually lead to hyperthyroidism by overproduction of thyroid-stimulating auto-antibodies. Specifically, HLA-DRB1 may predispose to an autoimmune-mediated reduction in insulin secretory function, either by a loss of beta cell mass or interference with early insulin release [27, 28]. Although not all schizophrenic patients suffer from glucose dysfunction, recent evidence has indicated a propensity toward such an association. Schoepf et al. [29] demonstrated that the prevalence of type 2 diabetes was increased in schizophrenia compared to hospitalized controls, while the findings of Perry et al. [30] suggests a potential link between pre-diabetic markers, in particular impaired glucose tolerance and insulin resistance, and first-episode psychosis.

Very few studies, to date, have examined the correlation between the structure of the HLA-DRB1 polymorphism and GD, schizophrenia, and T2D. A few studies have shown that an arginine residue at position 74 of the HLA-DRB1 chain plays an important role in the genetic susceptibility to those diseases [31, 32]. Since most DR3 subtypes contain DRb-Arg74 it was possible that the association of DRb-Arg74 with GD was merely a reflection of the association of DR3 with GD, schizophrenia, and T2D [31]. It is much more likely that the DR structure itself could explain some of the well-established DR3 associations with these autoimmune diseases.

## Conclusion

Our study suggests that the HLA DRB1*03 haplotype may explain a common etiology underlying the co-morbidity of Graves’ disease, type 2 diabetes, and schizophrenia.

## Abbreviations

GD: Graves’ disease
DT2: type 2 diabetes
HLA: human leukocyte antigen
